# Substrate Protection in Controlled Enzymatic Transformation of Peptides and Proteins

**DOI:** 10.1002/cbic.202100217

**Published:** 2021-06-14

**Authors:** Yan Zhao

**Affiliations:** ^1^ Department of Chemistry Iowa State University Ames IA 50011–3111 USA

**Keywords:** Binding, molecular imprinting, peptide, protection, proteolysis

## Abstract

Proteins are involved in practically every single biological process. The many enzymes involved in their synthesis, cleavage, and posttranslational modification (PTM) carry out highly specific tasks with no usage of protecting groups. Yet, the chemists’ strategy of protection/deprotection potentially can be highly useful, for example, when a specific biochemical reaction catalyzed by a broad‐specificity enzyme needs to be inhibited, during infection of cells by enveloped viruses, in the invasion and spread of cancer cells, and upon mechanistic investigation of signal‐transduction pathways. Doing so requires highly specific binding of peptide substrates in aqueous solution with biologically competitive affinities. Recent development of peptide‐imprinted cross‐linked micelles allows such protection and affords previously impossible ways of manipulating peptides and proteins in enzymatic transformations.

## Graphical abstract

**Highly specific enzymes** make protecting groups unnecessary in biology but some enzymes including proteases and kinases have multiple biological substrates and there is strong interest to stop a specific enzyme‐catalyzed reaction without inhibiting the enzyme itself. Recent developments in peptide‐imprinted nanoparticles allow precise protection of peptide substrates and open up previously impossible ways to manipulate enzymatic reactions.

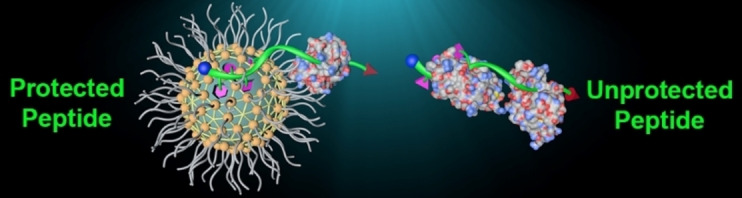

## Introduction

Enzymatic efficiency and selectivity represent the ultimate goals of chemists who seek to develop catalysts for their interested reactions. Indeed, under largely ambient conditions in neutral aqueous solutions, enzymes hydrolyze particular amide bonds, selectively oxidize hydrocarbons, convert nitrogen into ammonia, and perform all kinds of transformations vital to the biological world.

The high selectivity of enzymatic catalysis allows cells to carry out desired biochemical transformations from exceedingly complex mixtures without usage of any protecting groups. Glycosyltransferases and glycosidases, for example, effortlessly make complex glycans and cleave them at specific locations.^[1]^ In contrast, to synthesize even relatively simple glycans, chemists generally have to employ extensive protective/deprotective chemistry to deal with the many hydroxyl groups on the sugar building blocks that have little or no difference in intrinsic reactivity.^[2]^ Only in this year of 2021, synthetic catalysts appeared in the literature that could hydrolyze oligo‐ and polysaccharides with a reasonable level of selectivity.^[3]^

Protective groups have been an indispensable tool in modern organic chemistry, not only in the synthesis of biomolecules such as carbohydrates, peptides, and nucleic acids full of degenerate functional groups, but also in total synthesis of almost any complex, multifunctional molecules.^[4]^ Whenever chemists want to perform a chemical reaction that has compatibility issues with existing functional groups in the molecule, a straightforward and often the most reliable method is to protect the incompatible groups prior to the reaction and deprotect them at a suitable stage later on.

It seems, with the abundance of highly selective and even substrate‐specific enzymes, protection/deprotection is neither necessary nor useful in biology. However, this is not the case, at least when it comes for researchers to intervene and interrogate certain biological processes.

A good example is in the proteolysis of peptides and proteins. Cancer cells rely on over‐expressed proteases during their invasion and spread because of the need to remodel tissues.^[5]^ Since the same proteases may be used by normal cells to perform their cellular functions, traditional protease inhibitors tend to have high toxicity. Many enveloped viruses depend on a critical proteolytic activation step in their cellular infection including coronavirus,^[6]^ HIV‐1,^[7]^ and influenza virus A.^[7]^ Selective inhibition of a specific proteolytic reaction instead of all proteolysis is again vital to the antiviral treatment. Antibodies can be used to block proteolytic cleavage sites on proteins^[8]^ but they are expensive and fragile molecules made of polypeptides, which are susceptible to broad‐specificity proteases themselves.

One other example is in the post posttranslational modification (PTM) of proteins. Kinases catalyze phosphorylation of proteins, a reaction critical to numerous processes in cell signaling, regulation, and development.^[9]^ However, a vast number of potential phosphorylation sites exist in a cell, ∼700,000 by one estimation.^[9b]^ Even if most of these sites are buried and kinases have their own preferred substrates, a cell still has a large number of substrates for a given kinase.^[9]^ Traditional enzymatic inhibition is again facing a problem in this case, because unintended consequences will emerge when a multisubstrate kinase is shut down.^[10]^

A long‐recognized solution to the above problems lies in the selective inhibition of the peptide or protein substrates.^[11]^ If a particular substrate of a protease or kinase can be selectively protected from the enzyme, one would be able to shut down a specific biological reaction with high precision. Such protection can help researchers understand the biological ramifications of the masked biological reaction, useful in mechanistic biology and also potentially in disease treatment.^[8]^ Over the years, a few research groups have reported protection of peptides from chemical or enzymatic reactions, mainly using small‐molecule synthetic receptors. The reactions involved include proteolysis,[^11^, ^12^] acetylation of lysine side chains,^[13]^ tyrosine phosphorylation,^[14]^ and demethylation of methylated lysine side chains.^[15]^

## Molecular Recognition of Peptides

The scarcity of peptide protection in the literature points to a great need in peptide recognition, especially of complex biological peptides. To protect a peptide sequence from its enzymatic catalyst, one needs a receptor to bind the peptide with high affinity and selectivity in aqueous solution. Supramolecular chemistry in the last several decades largely have stayed in organic solvents where directional noncovalent forces such as hydrogen bonds are effective.^[16]^ Although examples of strong synthetic receptors in aqueous solution exist,^[17]^ they are exceptions rather than rules and a general strategy for effective molecular recognition of complex biological molecules in water is missing.^[18]^ For peptides, a particular challenge is in the distinction of the 20 possible side chains of a peptide, some of which differ minutely. Leucine (L) and isoleucine (I), for example, differ in the position of a single methyl group by one carbon. Glutamic acid (E) has one extra methylene than aspartic acid (D), and tyrosine (Y) has one extra hydroxyl in comparison to phenylalanine (F).

Chemists have developed many scaffolds to build peptide receptors,^[19]^ often focusing on specific residues with good supramolecular handles such as acidic and basic amino acids.^[20]^ Tryptophan (W) and phenylalanine are also popular targets because their aromatic side chains can fit into appropriate macrocycles^[21]^ such as cyclodextrins,^[22]^ cucurbiturils,^[23]^ or self‐assembled nanocages.^[24]^ Other interesting platforms include molecular tweezers and clips,^[25]^ pseudopeptidic cages,^[26]^ and gold nanoparticles that can be functionalized on the surface.^[27]^

Principles of complementarity and preorganization are the central dogma of supramolecular chemistry.[^16^, ^28^] It is impractical, however, to apply them in peptide recognition with a molecularly synthesized receptor. This is because, to bind a guest with multipoint noncovalent interactions, the host generally is larger than the guest and needs to possess a complementary, often concave‐shaped binding interface. For a biological peptide with 10 to 20 amino acid residues that have subtly different side chains, a complementary host, if constructed step‐by‐step, would be too difficult to design and synthesize.

A potential solution to the above problem comes from molecular imprinting, a simple and powerful method to create guest‐complementary binding sites in a cross‐linked polymer network.^[29]^ The method involves formation of a covalent or noncovalent complex between a template (often the guest molecule itself) and polymerizable functional monomers (FMs) in the presence of a large amount of a cross‐linker. Polymerization fixes the FMs around the template molecules in the polymer network. Cleavage of the covalent bonds between the FMs and the templates or, in the case of noncovalent imprinting, washing off the noncovalently trapped templates leaves behind “imprints” or guest‐shaped voids in the polymer. The FMs turn into binding groups in the imprinted sites during polymerization and can increase the selectivity and binding affinity for the template molecules during rebinding.

Many molecularly imprinted polymers (MIPs) have been created for peptides since the conception of the technique.^[30]^ One of the earliest examples of noncovalent imprinting used amino acid derivatives as templates.^[31]^ Traditional MIPs are insoluble polymeric materials. Nonetheless, when prepared under precipitation polymerization, MIP nanoparticles, 10–100 nm in size, are obtained that have great biological compatibility.^[32]^ Materials imprinted against mellittin (the major component of bee venom) in this way could be used to remove the toxin from the bloodstream of living mice.^[33]^ MIP nanoparticles can be prepared for hydrophilic peptides as well, if they are first functionalized with a fatty acid acyl chain and anchored at the interface of inverse microemulsion.^[34]^ One other way to produce water‐soluble imprinted materials is to perform imprinting on the surface of (diacetylene‐containing) vesicles, which after polymerization could report the binding event by fluorescence.^[35]^

## Micellar Imprinting of Peptides

To selectively protect a peptide under many biological settings, a 30–100 nm nanoparticle is probably still too large. Often times, it is a specific sequence of a long peptide to be protected in an enzymatic reaction, and the remaining peptide sequences could be part of a protein tertiary structure. Other times, a long peptide has several reaction sites and a specific site is to be protected while others remain accessible to their enzymatic catalysts. For all these situations, a high precision of protection is required that demands a peptide protector much smaller in size.

Our group in 2013 developed a method of molecular imprinting within doubly cross‐linked surfactant micelles.^[36]^ The so‐called molecularly imprinted nanoparticles (MINPs) are ∼5 nm in diameter with surface ligands and ∼4 nm without. They are, hence, similar to a medium‐sized protein in dimension and quite a bit smaller than typical antibodies (∼10 nm). Their surface charge can be tuned by different types of cross‐linkable surfactants.^[37]^ Micellar imprinting was first used to create selective receptors for bile salt derivatives and then quickly expanded to a wide range of biologically interesting small molecules/drugs,[^36^, ^37^, ^38^] carbohydrates,^[39]^ and peptides,^[40]^ *all in water*. Most recently, they are converted into artificial enzymes to catalyze a range of chemical reactions.[^3^, ^41^]

**Scheme 1:**
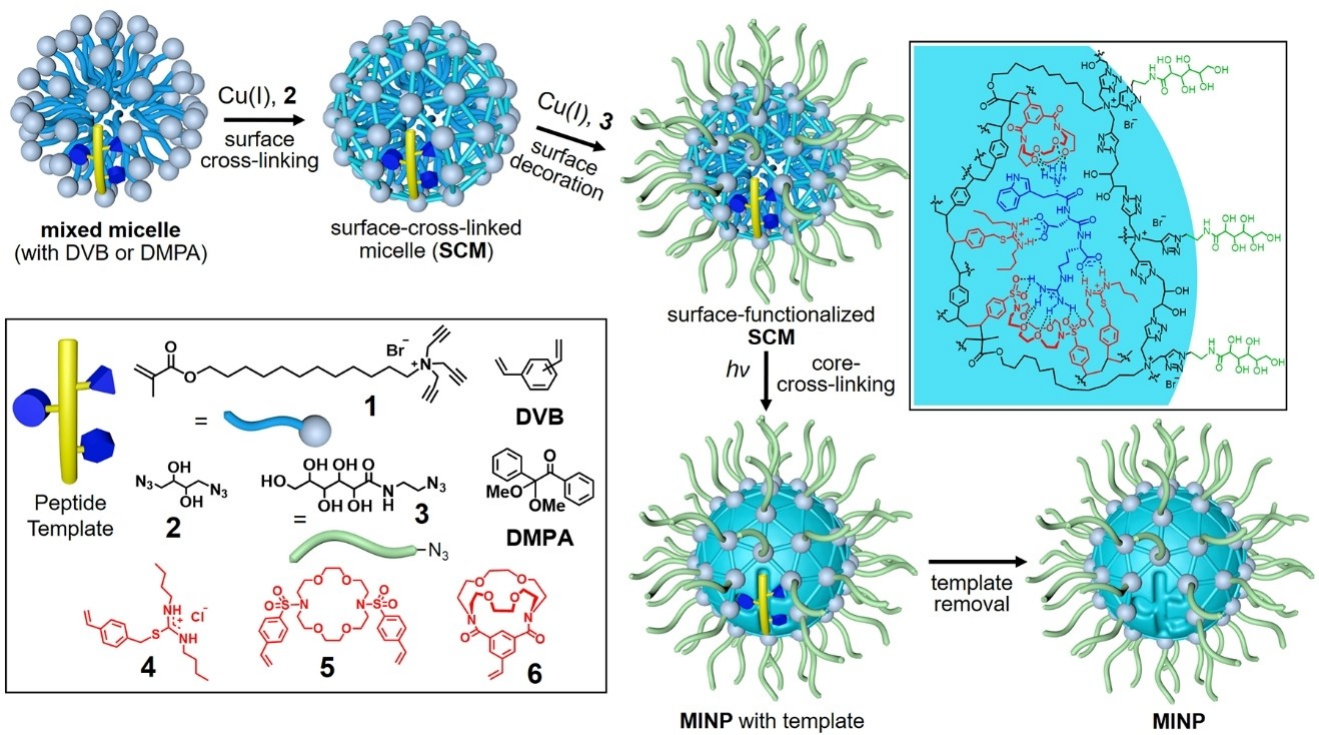
Preparation of peptide‐binding MINP from molecular imprinting of a cross‐linked micelle, with a schematic representation of the cross‐linked structure containing WDR bound by polymerized FMs. (Reprinted with permission from Ref. [51]. Copyright 2021, the American Chemical Society.)

As shown in Scheme 1, micellar imprinting starts with spontaneous formation of micelles using a cross‐linkable surfactant (**1**) in the presence of the interested peptide as the template molecule, divinylbenzene (DVB, a free‐radical cross‐linker), and a small amount of 2,2‐dimethoxy‐2‐phenylaceto‐phenone (DMPA, a photo initiator). The surface of the micelle is covered with a dense layer of alkyne groups from tripropargylammonium headgroup of the surfactant, and can be cross‐linked by diazide **2** in the presence of Cu(I) catalysts via the highly efficient click reaction. Another round of click reaction with monoazide **3** installs a layer of hydrophilic ligands on the surface of the micelle. (The sugar‐derived surface ligands are highly hydrophilic but insoluble in organic solvents such as acetone and methanol, and thus help the isolation and purification of the final MINPs.)

Micelles are highly dynamic, with diffusion‐controlled intermicellar exchange of surfactants.^[42]^ Covalently tethered on the surface, the surface‐cross‐linked micelle (SCM) becomes a nanoconfined space for molecular imprinting, as UV irradiation initiates free‐radical polymerization/cross‐linking around the template molecule in the micellar core.^[43]^ The nanoconfinement is found to be extremely important to the large imprinting factors obtained from micellar imprinting (often in the hundreds[^39d^, ^41c^] and sometimes up to 10,000^[40e]^). In addition, MINPs can easily detect the addition,^[43]^ removal,^[43]^ and shift^[40a]^ of a single methyl (or methylene) group in the guest binding.

For peptide binding, we initially focused on those rich in hydrophobic amino acids because they have a strong driving force to enter the micelle.^[40a]^ Our reasoning was that the hydrophobic side chains of amino acids have different degrees of hydrophobicity. For common hydrophobic amino acids, their side chains – shown schematically as blue shapes in Scheme 1 – differ in size, shape, and hydrophobicity. Thus, a “hydrophobic code” exists for each peptide that describes the number, size, shape, and distribution of its hydrophobic side chains.

Micellar imprinting, indeed, was found to create a complementary array of hydrophobic indentations or “dimples” on the cross‐linked micelles, essentially “encoding” the MINPs with supramolecular information to match the hydrophobic “code” of the peptide. These imprinted hydrophobic “dimples” turned out highly discriminating in their binding, to the point that the shift of a single methyl in leucine and isoleucine in isomeric di‐ and tripeptides could be distinguished, as well as phenylalanine and tyrosine.^[40a]^ The binding was also highly selective. When 5 MINPs were created for 5 biological peptides, negligible cross‐reactivity was observed when a particular peptide was titrated with the 4 nonmatching MINPs or, conversely, a particular MINP with the 4 nonmatching peptides.

Specific FMs (**4**–**6**) can be included in the MINP preparation to further improve the binding. They generally contain a polymerizable vinyl group and a molecular recognition motif targeting specific functional groups on the peptide (see the schematic representation of WDR bound by polymerized FMs in Scheme 1). FM **4**, for example, binds carboxylic acids through the hydrogen bond‐reinforced thiouronium–carboxylate salt bridge.^[40c]^ FM **5**, with abundant hydrogen‐bond acceptors in the structure, prefers the guanidinium side chain of arginine.^[40b]^ FMs **6** is selective for the amino group on the side chain of lysine and also on the N‐terminus.^[40d]^ With these FMs, we can target the hydrophobic, acidic, and basic groups of a peptide simultaneously, greatly enhancing both the binding selectivity and affinity of the MINP.^[40d]^ The functionalized MINPs have been shown to distinguish closely related hydrophilic residues such as aspartic acid/glutamic acid and lysine/arginine.

One might be surprised by how well these hydrogen‐bonded FMs work in MINP formation and binding. The mechanism is the same as how proteins and nucleic acids use these noncovalent forces in water – i. e., in a relatively nonpolar microenvironment where water is largely excluded. Although hydrogen‐bonds are weakened by strong solvent competition in an aqueous solution, they are much stronger in the hydrophobic core of a micelle.^[44]^

**Figure 1:**
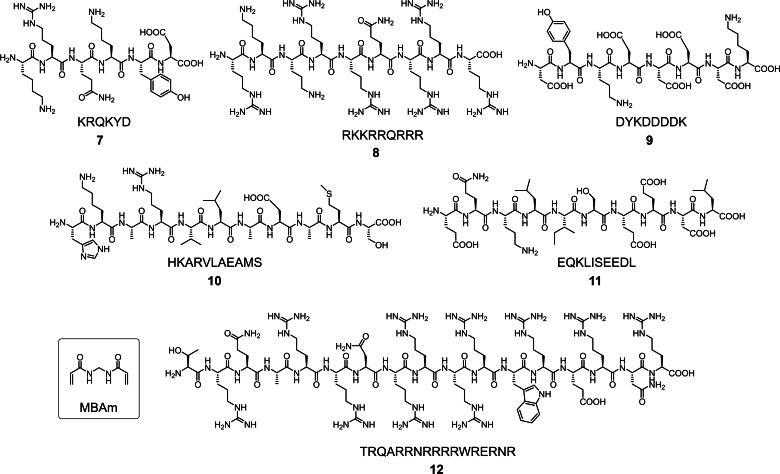
Structures of peptide templates used in the synthesis of MBAm‐functionalized MINPs.

Most recently, we discovered that, instead of specially designed FMs, commercially available amide‐containing cross‐linkers such as *N*,*N*′‐methylene‐bisacrylamide (MBAm) can be used instead of DVB during micellar imprinting (Figure 1).^[40e]^ The radical initiator (DMPA), being hydrophobic, strongly prefers to reside within the nonpolar core of the micelle. Once the initiating radical reacts with the methacrylate of the cross‐linkable surfactant (**1**) inside the SCM, the propagating radical is covalently attached to the micellar core and can polymerize only the MBAm molecules diffused to the palisade layer of the micelle. As a result, a belt of hydrogen‐bonding groups is formed near the surfactant/water interface, around the peptide template residing in the same area by its amphiphilicity.

**Figure 2:**
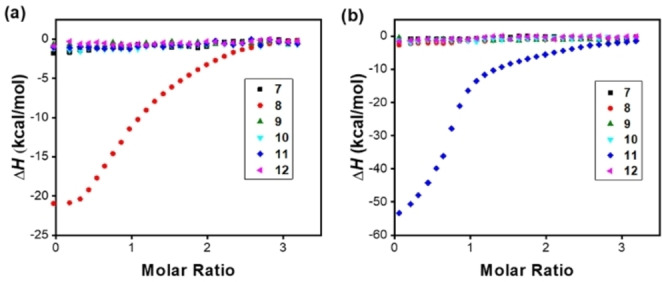
(a) ITC titration of peptides **7**–**12** to (a) MINP(**8**) and (b) MINP(**11**), showing only the desired peptide bound by the MINP. [MINP]=5.0 μM. [peptide]=75 μM in 10 mM HEPES buffer. The MINPs were prepared with 1 : 1 [**1**]/[MBAm]. (Reprinted with permission from Ref. [40e]. Copyright 2020, the American Chemical Society.)

When we compared the binding properties of MINPs prepared with DVB (our normal core‐cross‐linker) plus FMs and those prepared with MBAm (essentially as a hydrogen‐bonding functional cross‐linker), the MBAm‐cross‐linked MINPs were pleasantly found to outperform the DBV‐cross‐linked, functionalized MINPs consistently (Table 1). The binding constants for a number of complex biological peptides (**7**–**12** in Figure 1) ranged from 60 to 90×10^5^ M^−1^, corresponding to 110–170 nM of binding affinity. Excellent binding selectivity was also observed (Figure 2).^[40e]^

**Table 1:** Binding data for biological peptides **7**–**12** by MINPs prepared with DVB and FMs, and by MINPs prepared with MBAm without FMs.^[a]^

Entry	Template	Cross‐linker	*K*_a_ [×10^5^ M^−1^]	−Δ*G* [kcal/mol]	*N* ^[b]^
1	**7**	DVB	34.4±1.73	8.91	0.9±0.1
2		MBAm	62.2±2.32	9.26	0.9±0.1
3	**8**	DVB	45.3±2.85	9.07	1.1±0.1
4		MBAm	67.50±2.66	9.31	1.1±0.1
5	**9**	DVB	59.2±0.31	9.23	1.1±0.1
6		MBAm	73.10±2.47	9.36	1.2±0.1
7	**10**	DVB	82.3±2.29	9.43	0.9±0.1
8		MBAm	89.10±2.47	9.47	1.1±0.1
9	**11**	DVB	66.4±2.65	9.30	0.8±0.1
10		MBAm	72.50±1.27	9.35	0.9±0.1
11	**12**	DVB	53.40±1.84	9.17	1.1±0.1
12		MBAm	66.20±3.36	9.30	1.0±0.1

[a] The titrations were performed in HEPES buffer (10 mM, pH 7.4) in duplicates at 298 K and the errors between the runs were <10 %. For MINPs prepared with FMs, the following stoichiometry was used in the formulation: 1.5 : 1 for **4**/carboxylate, 1 : 1 for **6**/amine, and 1 : 1 for **5**/arginine. [b] *N* is the number of binding sites per nanoparticle determined by isothermal titration calorimetry (ITC). (Reprinted with permission from Ref. [40e]. Copyright 2020, the American Chemical Society.)

MINP contains hydrogen‐bonding groups including triazole, hydroxyl, and ester. Although these “background” interactions cannot be defined as precisely as the supramolecular “codes” defined by the specifically shaped hydrophobic dimples and the specially designed FMs, they are expected to be optimized to some extent during the imprinting process, for both the peptide backbone and side chains. These secondary interactions also can play important roles, evident from the binding of peptides containing glycine that lacks a side chain.[^40a^, ^45^]

## Sequence‐Selective Protection of Peptides by MINPs in Enzymatic Reactions

The nanodimension of MINPs and their strong and selective bindings for complex biological peptides bode well for their usage as protective agents for peptides. The Michaelis constants for common proteases are in the submillimolar to millimolar range^[46]^ and those for kinases range from micromolar to millimolar.^[47]^ The 100–200 nanomolar binding affinities (sometimes as low as 20 nM) of MINPs for hydrophobic and hydrophilic biological peptides^[40]^ suggest that selective binding should be totally achievable.

Our first model peptide for protected proteolysis was Angiotensin III (A‐III, RVYIHPF),^[48]^ cleavable by two common endopeptidases ‐trypsin after arginine (R) and by chymotrypsin after tyrosine (Y). LCMS analysis showed that MINP(A), i. e., MINP prepared with A‐III as the template, suppressed the proteolysis of the peptide to ≤10 % during a period of 2 h at 1 equiv. in the trypsin proteolysis and 2 equiv. in the chymotrypsin proteolysis. Nonimprinted nanoparticles (NINPs) prepared without templates only slowed down the reaction slightly. A strong correlation between binding and protection was observed when MINPs targeting the first 4, 5, and 6 amino acids of the *N*‐, and *C*‐terminal sequences were used for the protection. The protection factor, defined as the ratio between the yield in buffer at 2 h and the yield in the presence of the MINP, showed a linear relationship to the binding free energy.

Interestingly, the proteolytic yield of A‐III in the presence of MINP(A) fitted well to a 1 : 1 binding isotherm against the MINP concentration. The apparent “binding constant” obtained for trypsin inhibition (*K_a_* =(2.35±0.31)×10^7^ M^−1^) was quite close to the actual binding constant determined by ITC (*K_a_* =(1.89±0.13)×10^7^ M^−1^), suggesting the protection happened almost strictly with a 1 : 1 stoichiometry. Although the protection‐based “binding constant” for chymotrypsin was a few times lower than the ITC‐determined value, a strong binding–protection correlation was still observed. MINP protection was also found to work well for hydrophilic peptides (LRRASLG, PAGYLRRASVAQLT, and TGHGLRRSSKFCLK), if suitable FMs were used in the MINP preparation.

**Figure 3:**
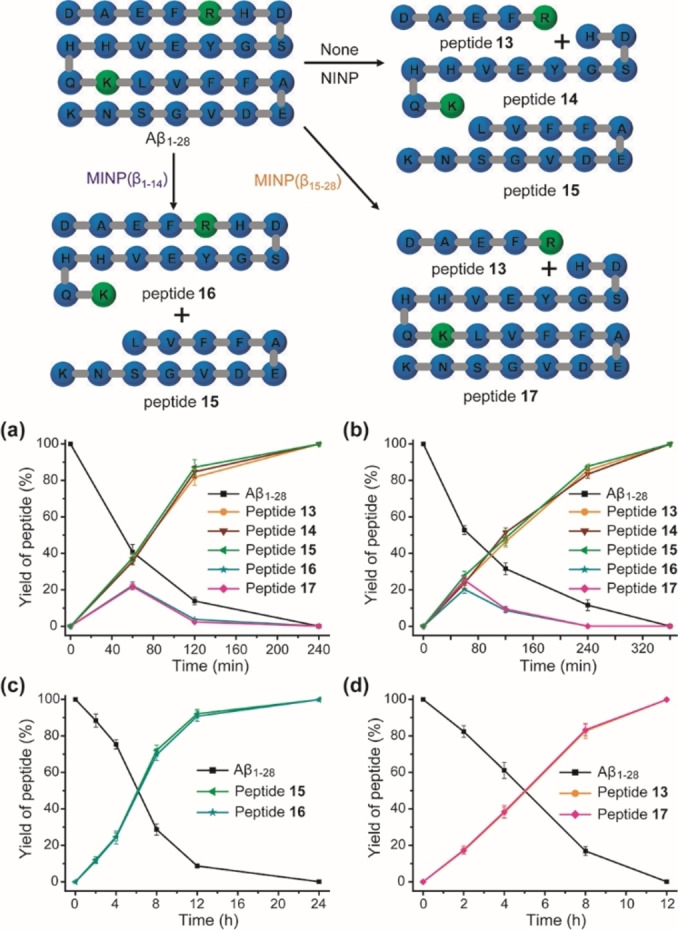
Product distribution curves in the trypsin digestion of Aβ_1–28_ in buffer (a) and in the presence of 1 equiv. NINP (b), MINP(β_1–14_) (c), and MINP(β_15–28_) (d). (Reprinted with permission from Ref. [48]. Copyright 2021, Wiley‐VCH.)

β‐Amyloid peptides are released through proteolysis and implicated in Alzheimer's disease.^[49]^ We decided to use Aβ_1–28_ to demonstrate selective protection of a fragment of a long peptide because it contains two cleavable sites by trypsin – arginine at AA5 and lysine at AA16 (marked in green in Figure 3). Two MINPs, MINP(β_1–14_) and MINP(β_15–28_), were prepared, targeting the first and second halves of the parent peptide. ITC showed that the two MINPs bound the parent peptide strongly in pH 7.4 phosphate buffer, with *K*_a_=1.97×10^7^ and 3.06×10^7^ M^−1^, respectively.

In the phosphate buffer (Figure 3a) or in the presence of NINPs (Figure 3b), trypsin hydrolyzed Aβ_1–28_ to afford the expected peptide products (**13**–**15**). In addition, two peptides (**16** and **17**), with only the arginine or lysine cleaved, showed transiently in the first 2 h of reaction time. NINPs slowed down the proteolysis somewhat but the product distribution curves were similar in shape as those in the buffer.

A totally different product distribution was obtained when Aβ_1–28_ was treated with trypsin in the presence of 1 equiv. MINP(β_1–14_) or MINP(β_15–28_). The formerly transiently observed **16** (Figure 3c) and **17** (Figure 3d) were produced continually depending on which MINP protector was employed.

MINP protection did slow down the proteolysis of the exposed site, especially if the site was close to the protected sequence. For example, lysine 16 in Aβ_1–28_ was only two residues away from the protected sequence of Aβ_1–14_; its (selective) proteolysis in the presence of MINP(β_1–14_) took ∼24 h to complete (Figure 3c), instead of 4 h in buffer (Figure 3a) and 6 h with NINP (Figure 3b). Arginine 5, on the other hand, was 9 residues away from Aβ_15–28_ bound by MINP(β_15–28_) and its (selective) hydrolysis in Aβ_1–28_ took approximately 12 h (Figure 3d).

**Figure 4:**
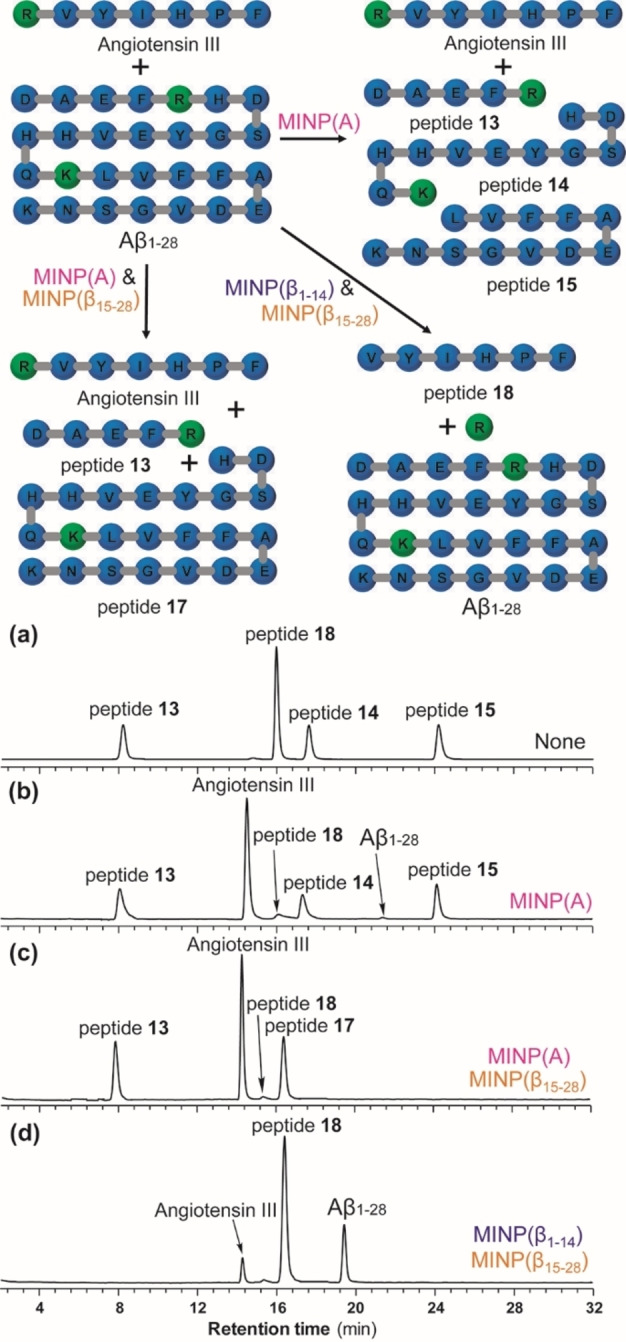
HPLC chromatograms of trypsin digestion of a 2 : 1 mixture of Angiotensin III and Aβ_1–28_ by trypsin (a) without any protection, and in the presence of (b) MINP(A), (c) MINP(A) & MINP(β_15–28_), and (d) MINP(β_1–14_) & MINP(β_15–28_). Reaction time was 4 h except in (c) which required 12 h for the selective hydrolysis of Aβ_1–28_. (Reprinted with permission from Ref. [48]. Copyright 2021, Wiley‐VCH.)

For MINP(A), MINP(β_1–14_), and MINP(β_15–28_), the nontemplating peptides showed very low cross‐reactivities (0.06–0.13 %) in the binding. This feature allowed us to carry out more advanced protections, using a 2 : 1 mixture of A‐III and Aβ_1–28_ for a proof of concept. Without any protector, the peptide mixture were digested by trypsin to afford peptides **13**–**15**, as well as **18** from A‐III (Figure 4a). One equivalent of MINP(A) largely suppressed the proteolysis of A‐III, while Aβ_1–28_ hydrolyzed (Figure 4b). If MINP(β_15–28_) was used together with MINP(A), Aβ_1–28_ underwent the anticipated selective cleavage after arginine 5 to afford **13** and **17** while A‐III stayed largely intact (Figure 4c). Most interestingly, MINP(β_1–14_) and MINP(β_15–28_) could shied the long Aβ_1–28_ together: after 4 h of reaction time, nearly 90 % of A‐III hydrolyzed in the mixture while Aβ_1–28_ persisted (Figure 4d). ITC confirmed that the long peptide indeed could bind both MINPs simultaneously, although the binding constants were several times lower than those measured with only one MINP, suggesting some steric/electrostatic repulsion existed when two MINPs came together to bind one long peptide.

Because the inhibition of the enzymatic reaction is driven strictly by selective binding, we expect that, anytime a peptide is bound more strongly by an MINP receptor than its enzyme catalyst, the enzymatic reaction can be inhibited. The prediction was confirmed recently in selective phosphorylation of peptide mixtures by cyclic AMP‐dependent protein kinase (PKA), an enzyme with over 100 physiological substrates.^[50]^ A particular challenge in controlled phosphorylation is that different substrates of a kinase generally have very similar or even identical “consensus motifs” surrounding the phosphorylation sites.^[50b]^ PKA, for example, phosphorylates peptides with an RRXS motif (X=a variable amino acid). Yet, MINP was able to control the PKA‐catalyzed phosphorylation of Kemptide (LRRASLG), β_2_‐adrenergic receptor peptide (TGHGLRRSSKFCLK), pyruvate kinase peptide (PAGYLRRASVAQLT), and cardiac myosin binding protein‐C peptide (FRRTSLAGGGRRISDSHE) completely.^[51]^ Note that Kemptide and pyruvate kinase peptide share identical consensus motifs, even the leucine (L) in front of the recognition motif. For cardiac myosin‐binding protein‐C peptide, selective protection of a fragment of the long peptide and cooperative protection of the entire sequence by two MINPs were both achieved.

**Figure 5:**
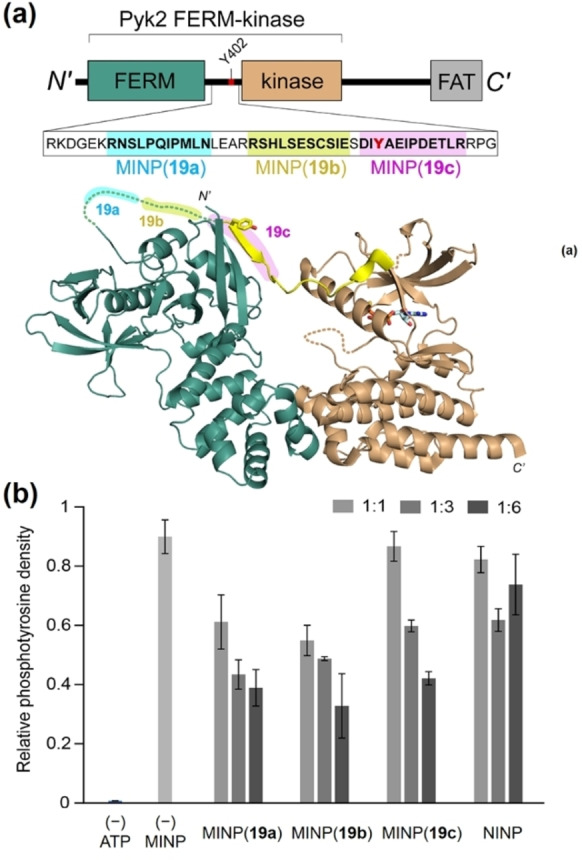
(a) Domain organization of Pyk2 and structural model depicting the Pyk2 FERM (PDB 4eku) and kinase (PDB 3fzp) aligned to the FAK FERM‐kinase (PDB 2j0j). The FAK FERM‐kinase linker is superimposed (yellow) to illustrate putative MINP binding sites. (b) Inhibition of Pyk2 autophosphorylation by MINPs at 1 : 1, 1 : 3, and 1 : 6 protein/MINP ratios, with the NINP as the control. [Pyk2]=1.0 μM. (Reprinted with permission from Ref. [51]. Copyright 2021, the American Chemical Society.)

Biological phosphorylation frequently occurs within a protein complex. One such example is the phosphotransfer step in the activation of the proline‐rich tyrosine kinase 2 (Pyk2), a regulator of leukocyte motility, bone remodeling, and neuronal development.^[52]^ As shown in Figure 5a, the Pyk2 activation occurs when tyrosine Y402 in the linker between the regulatory FERM and the kinase domain is autophosphorylated.^[53]^ The intramolecular nature of the reaction makes it even more challenging to protect the substrate because MINP binding will have to compete with intramolecular protein–protein interactions.

Since we can prepare MINPs conveniently to target different sections of a long peptide, we can essentially “scan” the linker by different MINPs and observe how the MINP binding affects the phosphorylation. Interestingly, when three MINPs targeting AA373–383, 388–398, and 400–411 of the linker, were added to the protein complex and ATP mixture, MINP(**19 a**) and MINP(**19 b**) turned out significantly more potent than MINP(**19 c**) in the inhibition of the autophosphorylation (Figure 5b), even though it was MINP(**19 c**) that directly impinged on Y402 in its binding. This unusual behavior might have resulted from a lower accessibility of **19 c** sequence, since evidence exists that suggests the autophosphorylation site could be part of an abbreviated β sheet.^[54]^

## Conclusions and Outlook

MINPs have a remarkable ability to bind complex biological peptides in aqueous solution. With appropriate functional monomers^[40d]^ and/or cross‐linkers,^[40e]^ they can frequently achieve tens of nanomolar binding affinities for peptides with 10–20 amino acid residues. Their ability to distinguish closely related residues including leucine/isoleucine,^[40a]^ phenylalanine/tyrosine,^[40a]^ glutamic acid/aspartic acid,^[40c]^ and lysine/arginine^[40b]^ makes them an extremely attractive class of materials for biological applications. Once the cross‐linkable surfactant, cross‐linker, and templates are available, their preparation takes less than 2 days and purification requires nothing other than precipitation and washing.

Because most enzymes bind their substrates with millimolar to micromolar affinities, MINPs are expected to compete effectively with many enzymes in the binding and, in turn, to shield their peptide substrates from enzymatic actions. Controlled proteolysis and phosphorylation are just examples chosen to illustrate the power of substrate protection, which should be quite general. Biology historically has been a great source of inspiration to chemists in their development of methods to recognize, transport, and transform molecules. Protection/deprotection, on the other hand, is largely an invention of chemists for the synthesis of complex organic molecules. Maybe, the strategy now is ready to find its way back into biology, as a way to return the favor.

## Conflict of interest

The authors declare no conflict of interest.
